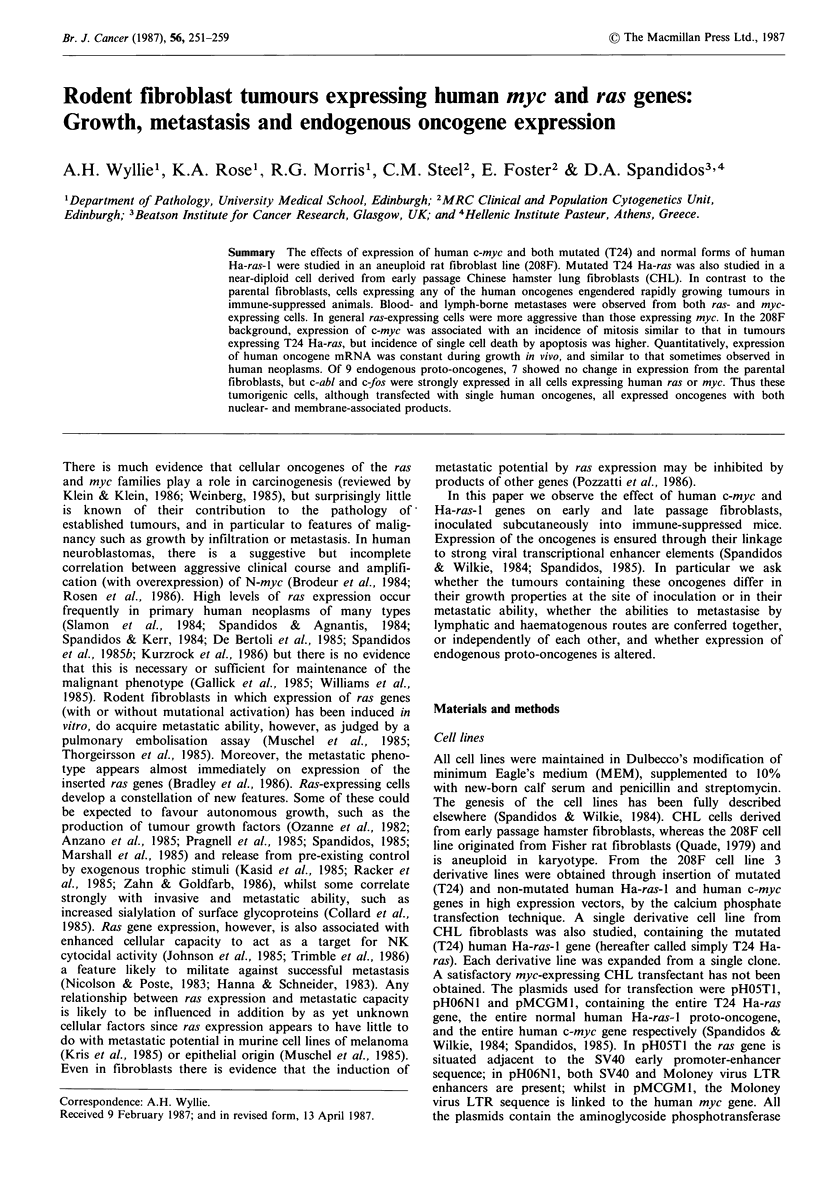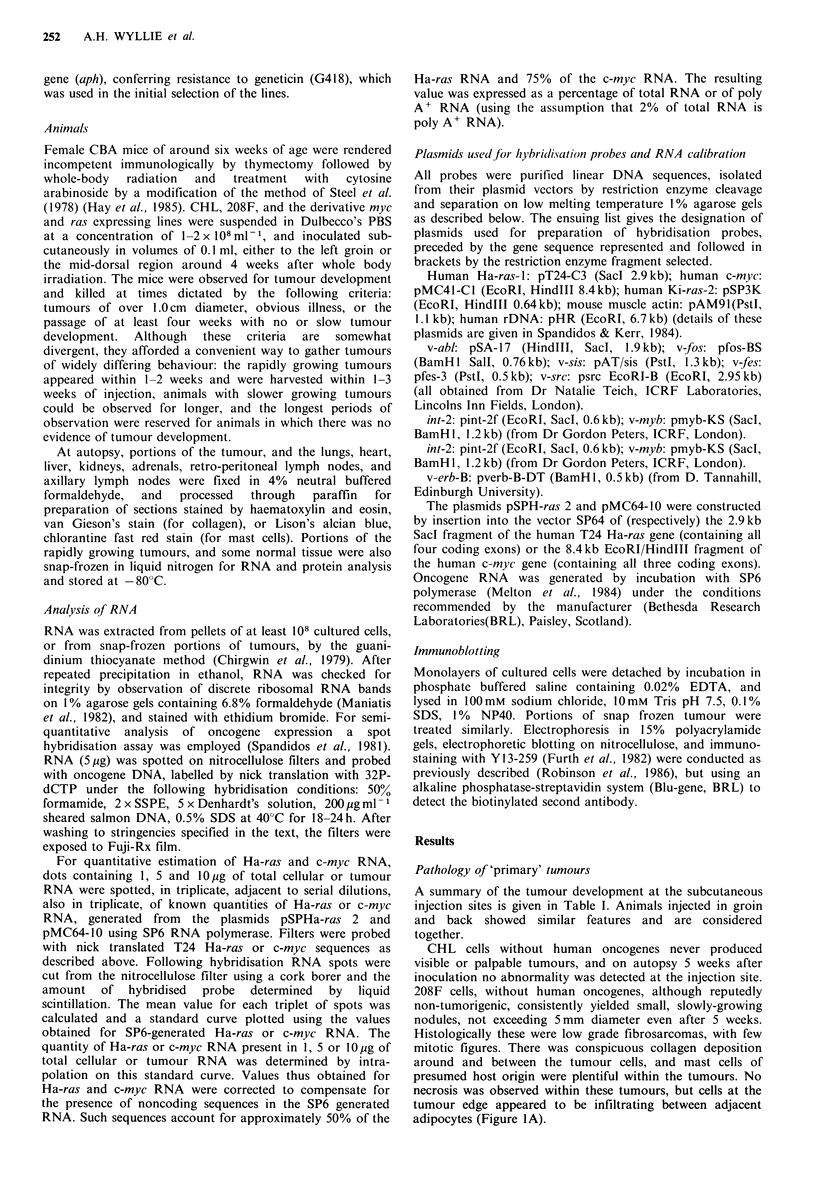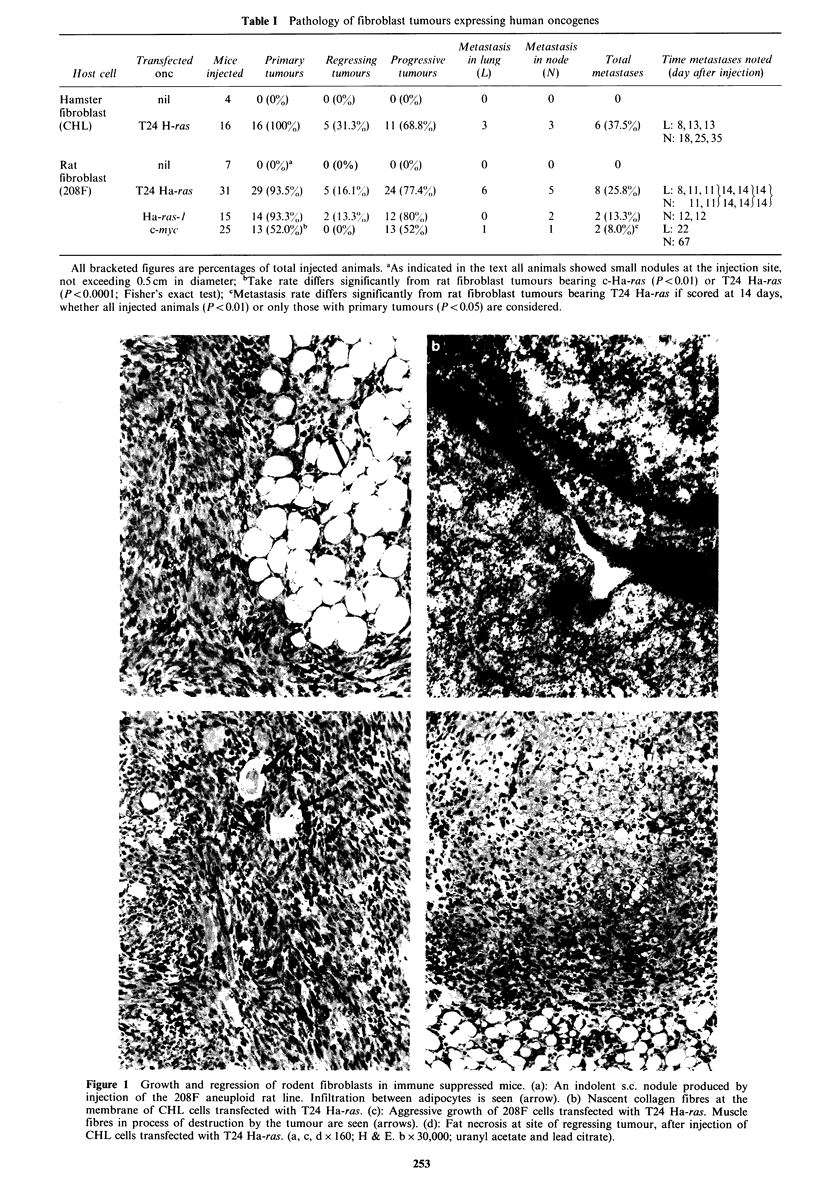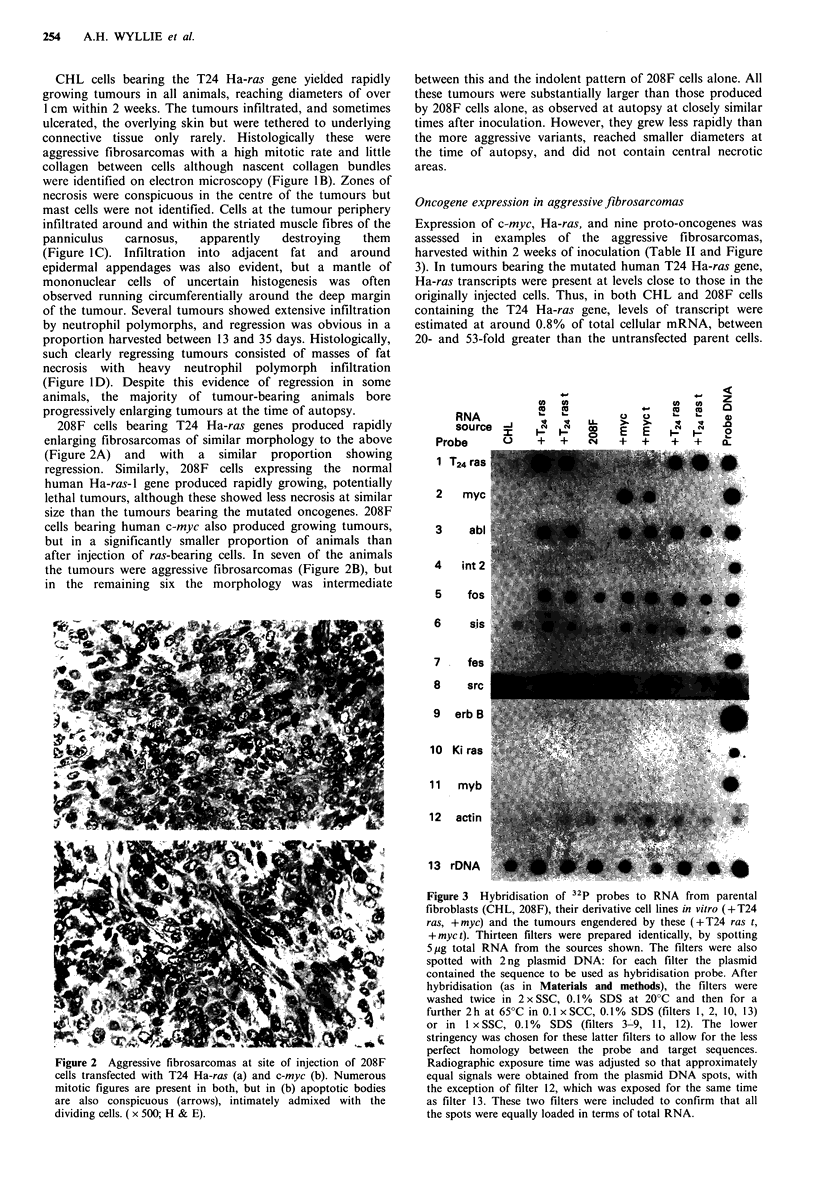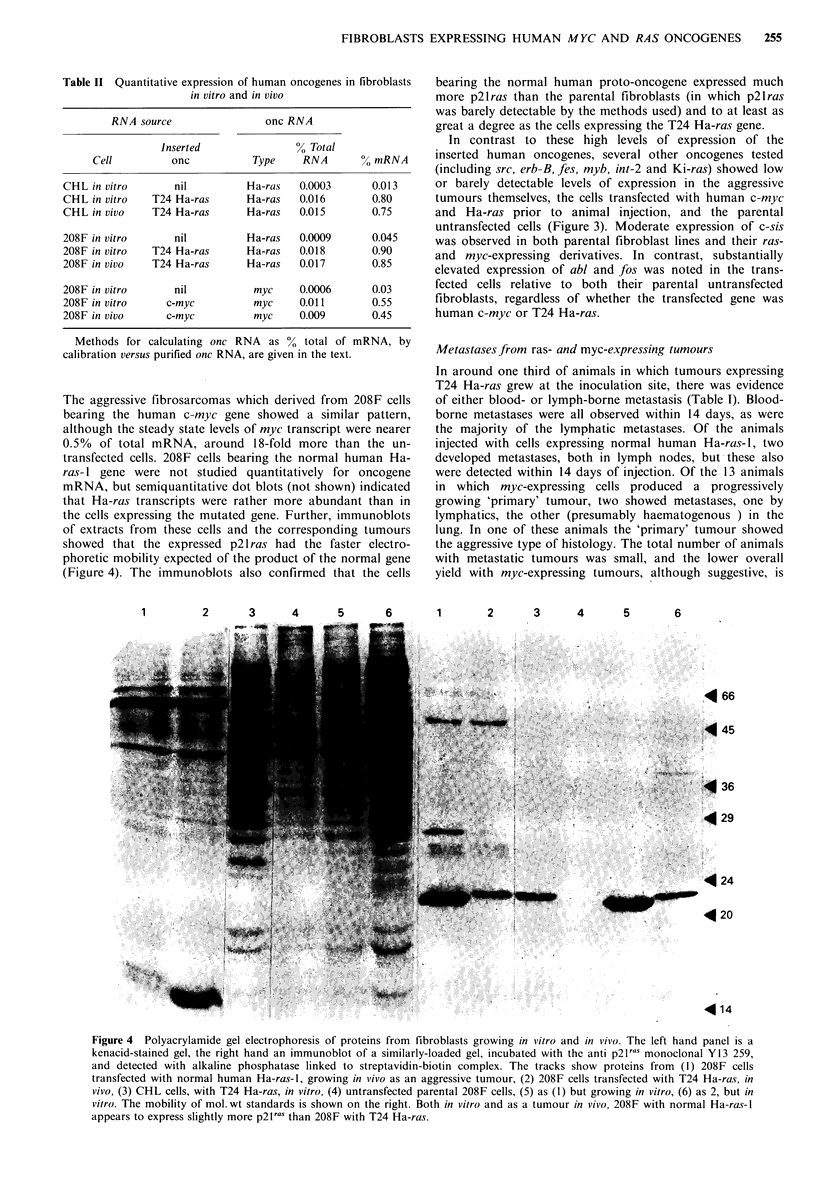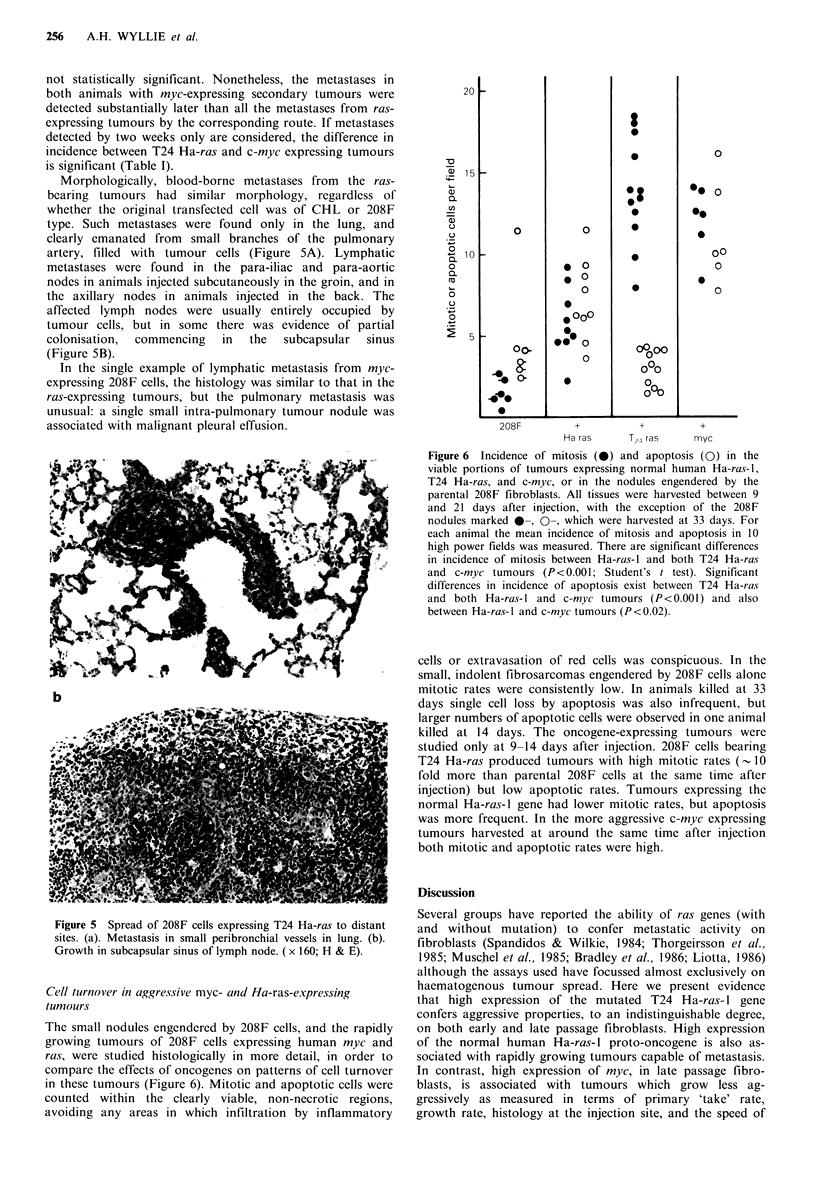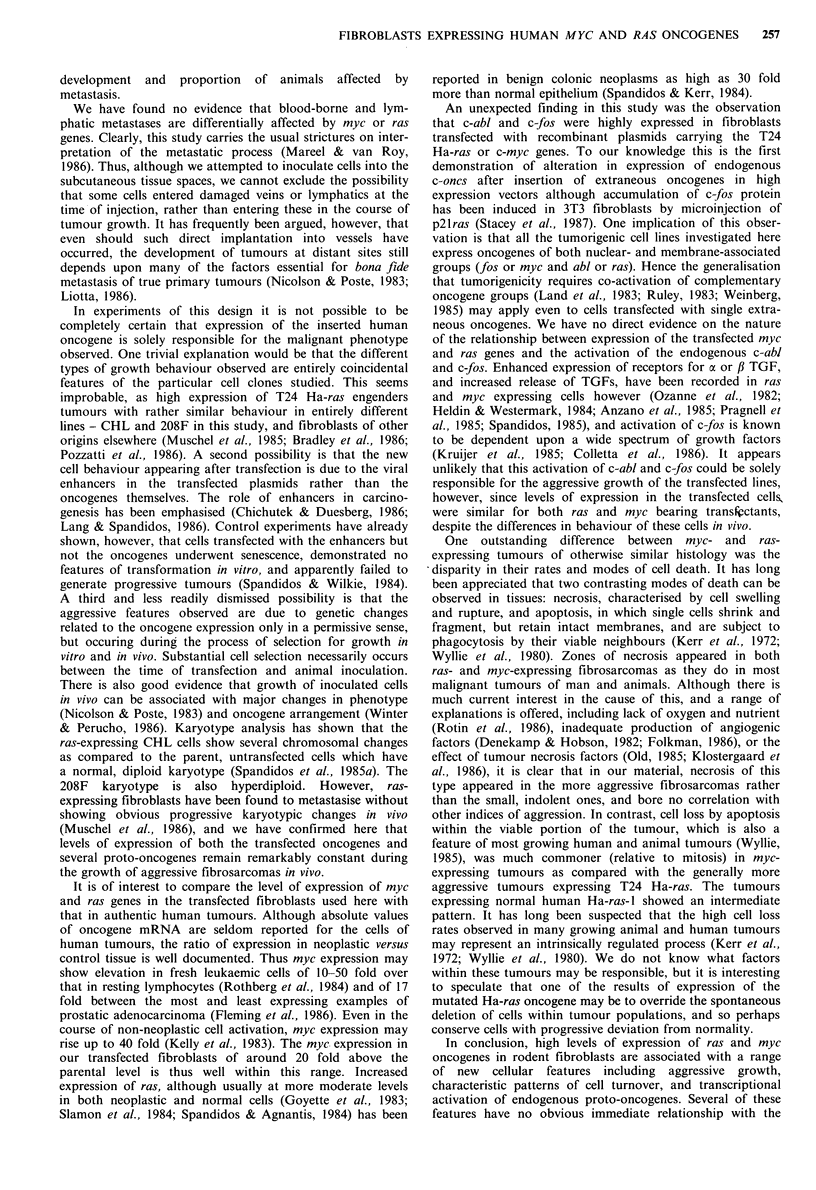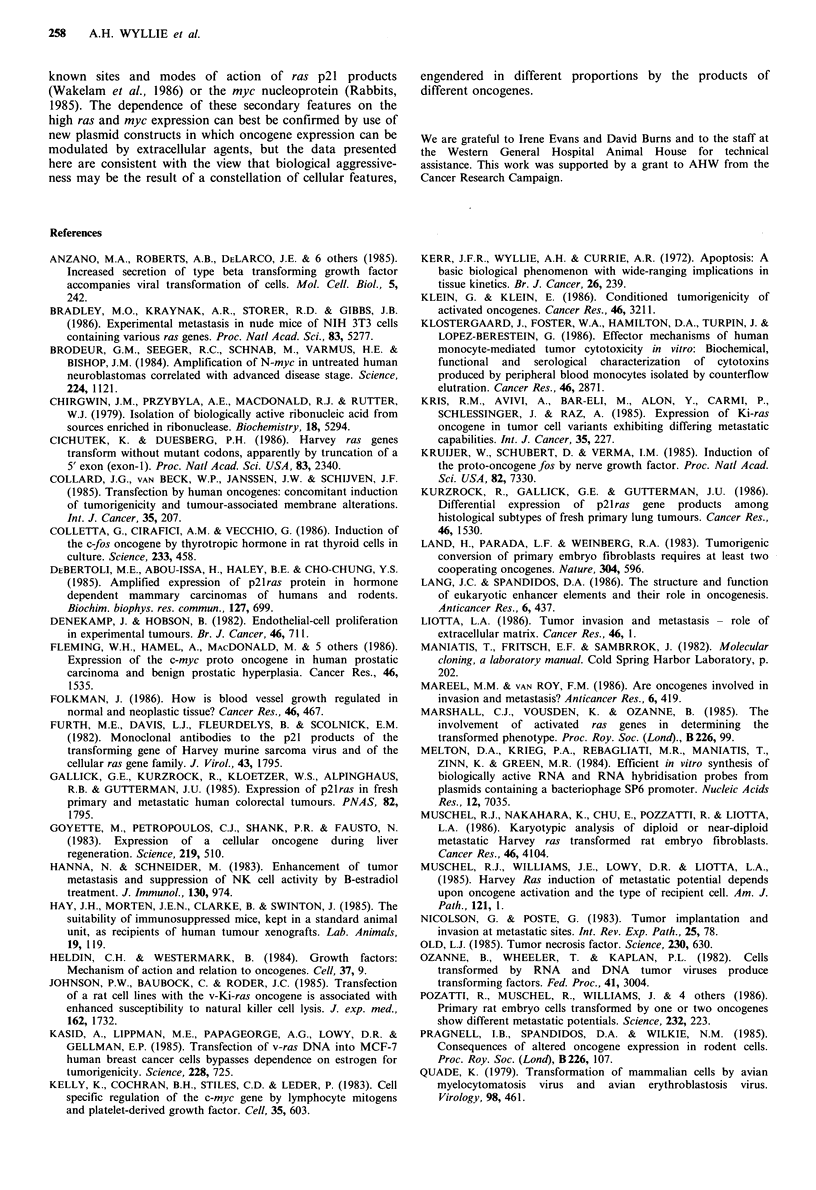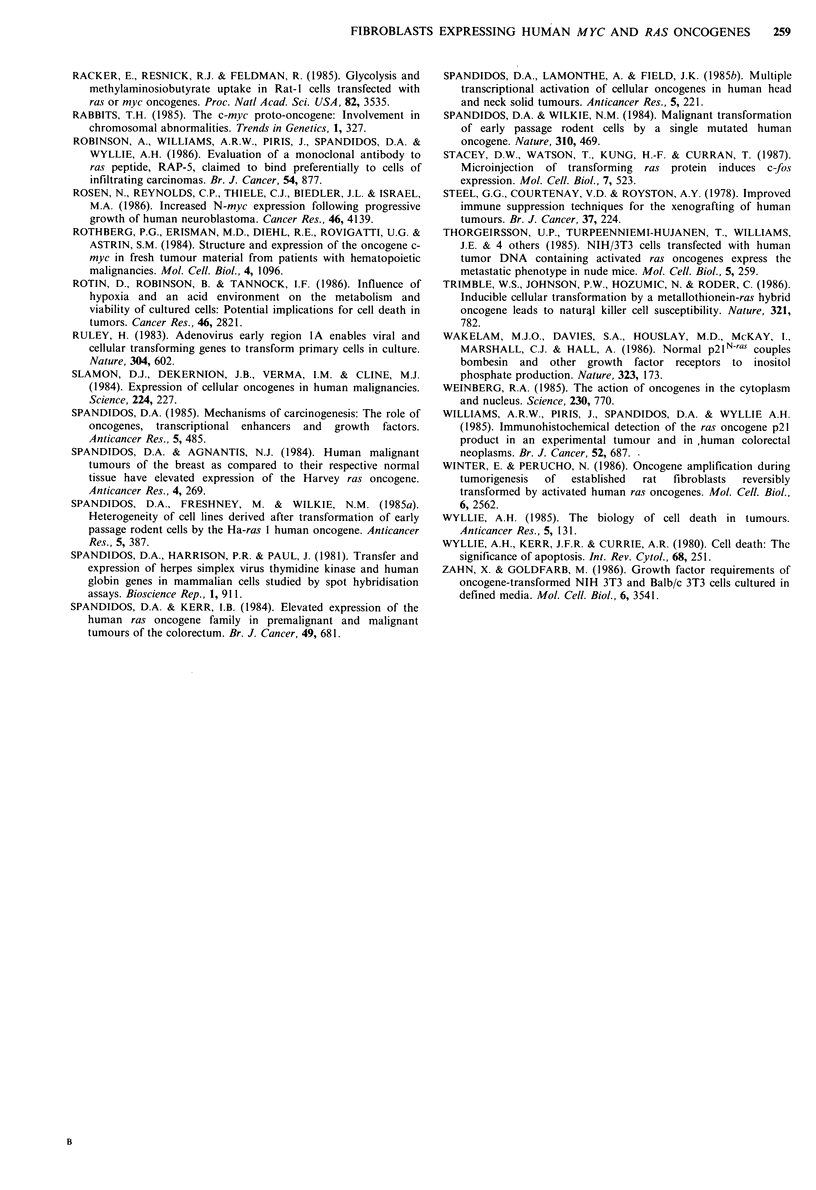# Rodent fibroblast tumours expressing human myc and ras genes: growth, metastasis and endogenous oncogene expression.

**DOI:** 10.1038/bjc.1987.186

**Published:** 1987-09

**Authors:** A. H. Wyllie, K. A. Rose, R. G. Morris, C. M. Steel, E. Foster, D. A. Spandidos

**Affiliations:** Department of Pathology, University Medical School, Edinburgh, UK.

## Abstract

**Images:**


Br. J. Cancer (1987), 56, 251–259 © The Macmillan Press Ltd., 1987

### Rodent fibroblast tumours expressing human *myc* and *ras* genes: Growth, metastasis and endogenous oncogene expression

A.H. Wyllie[1], K.A. Rose[1], R.G. Morris[1], C.M. Steel[2], E. Foster[2] & D.A. Spandidos[3,4]

[1]*Department of Pathology, University Medical School, Edinburgh;* [2]*MRC Clinical and Population Cytogenetics Unit, Edinburgh;* [3]*Beatson Institute for Cancer Research, Glasgow, UK; and* [4]*Hellenic Institute Pasteur, Athens, Greece.*

**Summary** The effects of expression of human c-*myc* and both mutated (T24) and normal forms of human Ha-*ras*-1 were studied in an aneuploid rat fibroblast line (208F). Mutated T24 Ha-*ras* was also studied in a near-diploid cell derived from early passage Chinese hamster lung fibroblasts (CHL). In contrast to the parental fibroblasts, cells expressing any of the human oncogenes engendered rapidly growing tumours in immune-suppressed animals. Blood- and lymph-borne metastases were observed from both *ras*- and *myc*-expressing cells. In general *ras*-expressing cells were more aggressive than those expressing *myc*. In the 208F background, expression of c-*myc* was associated with an incidence of mitosis similar to that in tumours expressing T24 Ha-*ras*, but incidence of single cell death by apoptosis was higher. Quantitatively, expression of human oncogene mRNA was constant during growth *in vivo*, and similar to that sometimes observed in human neoplasms. Of 9 endogenous proto-oncogenes, 7 showed no change in expression from the parental fibroblasts, but c-*abl* and c-*fos* were strongly expressed in all cells expressing human *ras* or *myc*. Thus these tumorigenic cells, although transfected with single human oncogenes, all expressed oncogenes with both nuclear- and membrane-associated products.

There is much evidence that cellular oncogenes of the *ras* and *myc* families play a role in carcinogenesis (reviewed by Klein & Klein, 1986; Weinberg, 1985), but surprisingly little is known of their contribution to the pathology of established tumours, and in particular to features of malignancy such as growth by infiltration or metastasis. In human neuroblastomas, there is a suggestive but incomplete correlation between aggressive clinical course and amplification (with overexpression) of N-*myc* (Brodeur *et al.*, 1984; Rosen *et al.*, 1986). High levels of *ras* expression occur frequently in primary human neoplasms of many types (Slamon *et al.*, 1984; Spandidos & Agnantis, 1984; Spandidos & Kerr, 1984; De Bertoli *et al.*, 1985; Spandidos *et al.*, 1985*b*; Kurzrock *et al.*, 1986) but there is no evidence that this is necessary or sufficient for maintenance of the malignant phenotype (Gallick *et al.*, 1985; Williams *et al.*, 1985). Rodent fibroblasts in which expression of *ras* genes (with or without mutational activation) has been induced *in vitro*, do acquire metastatic ability, however, as judged by a pulmonary embolisation assay (Muschel *et al.*, 1985; Thorgeirsson *et al.*, 1985). Moreover, the metastatic phenotype appears almost immediately on expression of the inserted *ras* genes (Bradley *et al.*, 1986). *Ras*-expressing cells develop a constellation of new features. Some of these could be expected to favour autonomous growth, such as the production of tumour growth factors (Ozanne *et al.*, 1982; Anzano *et al.*, 1985; Pragnell *et al.*, 1985; Spandidos, 1985; Marshall *et al.*, 1985) and release from pre-existing control by exogenous trophic stimuli (Kasid *et al.*, 1985; Racker *et al.*, 1985; Zahn & Goldfarb, 1986), whilst some correlate strongly with invasive and metastatic ability, such as increased sialylation of surface glycoproteins (Collard *et al.*, 1985). *Ras* gene expression, however, is also associated with enhanced cellular capacity to act as a target for NK cytocidal activity (Johnson *et al.*, 1985; Trimble *et al.*, 1986) a feature likely to militate against successful metastasis (Nicolson & Poste, 1983; Hanna & Schneider, 1983). Any relationship between *ras* expression and metastatic capacity is likely to be influenced in addition by as yet unknown cellular factors since *ras* expression appears to have little to do with metastatic potential in murine cell lines of melanoma (Kris *et al.*, 1985) or epithelial origin (Muschel *et al.*, 1985). Even in fibroblasts there is evidence that the induction of metastatic potential by *ras* expression may be inhibited by products of other genes (Pozzatti *et al.*, 1986).

In this paper we observe the effect of human c-*myc* and Ha-*ras*-1 genes on early and late passage fibroblasts, inoculated subcutaneously into immune-suppressed mice. Expression of the oncogenes is ensured through their linkage to strong viral transcriptional enhancer elements (Spandidos & Wilkie, 1984; Spandidos, 1985). In particular we ask whether the tumours containing these oncogenes differ in their growth properties at the site of inoculation or in their metastatic ability, whether the abilities to metastasise by lymphatic and haematogenous routes are conferred together, or independently of each other, and whether expression of endogenous proto-oncogenes is altered.

### Materials and methods

### *Cell lines*

All cell lines were maintained in Dulbecco's modification of minimum Eagle's medium (MEM), supplemented to 10% with new-born calf serum and penicillin and streptomycin. The genesis of the cell lines has been fully described elsewhere (Spandidos & Wilkie, 1984). CHL cells derived from early passage hamster fibroblasts, whereas the 208F cell line originated from Fisher rat fibroblasts (Quade, 1979) and is aneuploid in karyotype. From the 208F cell line 3 derivative lines were obtained through insertion of mutated (T24) and non-mutated human Ha-*ras*-1 and human c-*myc* genes in high expression vectors, by the calcium phosphate transfection technique. A single derivative cell line from CHL fibroblasts was also studied, containing the mutated (T24) human Ha-*ras*-1 gene (hereafter called simply T24 Ha-*ras*). Each derivative line was expanded from a single clone. A satisfactory *myc*-expressing CHL transfectant has not been obtained. The plasmids used for transfection were pH05T1, pH06N1 and pMCGM1, containing the entire T24 Ha-*ras* gene, the entire normal human Ha-*ras*-1 proto-oncogene, and the entire human c-*myc* gene respectively (Spandidos & Wilkie, 1984; Spandidos, 1985). In pH05T1 the *ras* gene is situated adjacent to the SV40 early promoter-enhancer sequence; in pH06N1, both SV40 and Moloney virus LTR enhancers are present; whilst in pMCGM1, the Moloney virus LTR sequence is linked to the human *myc* gene. All the plasmids contain the aminoglycoside phosphotransferase

Correspondence: A.H. Wyllie.
Received 9 February 1987; and in revised form, 13 April 1987.

gene (*aph*), conferring resistance to geneticin (G418), which was used in the initial selection of the lines.

*Animals*

Female CBA mice of around six weeks of age were rendered incompetent immunologically by thymectomy followed by whole-body radiation and treatment with cytosine arabinoside by a modification of the method of Steel *et al.* (1978) (Hay *et al.*, 1985). CHL, 208F, and the derivative *myc* and *ras* expressing lines were suspended in Dulbecco's PBS at a concentration of $1\text{–}2 \times 10^8\,\text{ml}^{-1}$, and inoculated subcutaneously in volumes of 0.1 ml, either to the left groin or the mid-dorsal region around 4 weeks after whole body irradiation. The mice were observed for tumour development and killed at times dictated by the following criteria: tumours of over 1.0 cm diameter, obvious illness, or the passage of at least four weeks with no or slow tumour development. Although these criteria are somewhat divergent, they afforded a convenient way to gather tumours of widely differing behaviour: the rapidly growing tumours appeared within 1–2 weeks and were harvested within 1–3 weeks of injection, animals with slower growing tumours could be observed for longer, and the longest periods of observation were reserved for animals in which there was no evidence of tumour development.

At autopsy, portions of the tumour, and the lungs, heart, liver, kidneys, adrenals, retro-peritoneal lymph nodes, and axillary lymph nodes were fixed in 4% neutral buffered formaldehyde, and processed through paraffin for preparation of sections stained by haematoxylin and eosin, van Gieson's stain (for collagen), or Lison's alcian blue, chlorantine fast red stain (for mast cells). Portions of the rapidly growing tumours, and some normal tissue were also snap-frozen in liquid nitrogen for RNA and protein analysis and stored at −80°C.

*Analysis of RNA*

RNA was extracted from pellets of at least $10^8$ cultured cells, or from snap-frozen portions of tumours, by the guanidinium thiocyanate method (Chirgwin *et al.*, 1979). After repeated precipitation in ethanol, RNA was checked for integrity by observation of discrete ribosomal RNA bands on 1% agarose gels containing 6.8% formaldehyde (Maniatis *et al.*, 1982), and stained with ethidium bromide. For semi-quantitative analysis of oncogene expression a spot hybridisation assay was employed (Spandidos *et al.*, 1981). RNA (5 μg) was spotted on nitrocellulose filters and probed with oncogene DNA, labelled by nick translation with 32P-dCTP under the following hybridisation conditions: 50% formamide, 2 × SSPE, 5 × Denhardt's solution, $200\,\mu\text{g ml}^{-1}$ sheared salmon DNA, 0.5% SDS at 40°C for 18–24 h. After washing to stringencies specified in the text, the filters were exposed to Fuji-Rx film.

For quantitative estimation of Ha-*ras* and c-*myc* RNA, dots containing 1, 5 and 10 μg of total cellular or tumour RNA were spotted, in triplicate, adjacent to serial dilutions, also in triplicate, of known quantities of Ha-*ras* or c-*myc* RNA, generated from the plasmids pSPHa-*ras* 2 and pMC64-10 using SP6 RNA polymerase. Filters were probed with nick translated T24 Ha-*ras* or c-*myc* sequences as described above. Following hybridisation RNA spots were cut from the nitrocellulose filter using a cork borer and the amount of hybridised probe determined by liquid scintillation. The mean value for each triplet of spots was calculated and a standard curve plotted using the values obtained for SP6-generated Ha-*ras* or c-*myc* RNA. The quantity of Ha-*ras* or c-*myc* RNA present in 1, 5 or 10 μg of total cellular or tumour RNA was determined by intrapolation on this standard curve. Values thus obtained for Ha-*ras* and c-*myc* RNA were corrected to compensate for the presence of noncoding sequences in the SP6 generated RNA. Such sequences account for approximately 50% of the Ha-*ras* RNA and 75% of the c-*myc* RNA. The resulting value was expressed as a percentage of total RNA or of poly $A^+$ RNA (using the assumption that 2% of total RNA is poly $A^+$ RNA).

*Plasmids used for hybridisation probes and RNA calibration*

All probes were purified linear DNA sequences, isolated from their plasmid vectors by restriction enzyme cleavage and separation on low melting temperature 1% agarose gels as described below. The ensuing list gives the designation of plasmids used for preparation of hybridisation probes, preceded by the gene sequence represented and followed in brackets by the restriction enzyme fragment selected.

Human Ha-*ras*-1: pT24-C3 (SacI 2.9 kb); human c-*myc*: pMC41-C1 (EcoRI, HindIII 8.4 kb); human Ki-*ras*-2: pSP3K (EcoRI, HindIII 0.64 kb); mouse muscle actin: pAM91(PstI, 1.1 kb); human rDNA: pHR (EcoRI, 6.7 kb) (details of these plasmids are given in Spandidos & Kerr, 1984).

v-*abl*: pSA-17 (HindIII, SacI, 1.9 kb); v-*fos*: pfos-BS (BamH1 SalI, 0.76 kb); v-*sis*: pAT/sis (PstI, 1.3 kb); v-*fes*: pfes-3 (PstI, 0.5 kb); v-*src*: psrc EcoRI-B (EcoRI, 2.95 kb) (all obtained from Dr Natalie Teich, ICRF Laboratories, Lincolns Inn Fields, London).

*int*-2: pint-2f (EcoRI, SacI, 0.6 kb); v-*myb*: pmyb-KS (SacI, BamH1, 1.2 kb) (from Dr Gordon Peters, ICRF, London).

*int*-2: pint-2f (EcoRI, SacI, 0.6 kb); v-*myb*: pmyb-KS (SacI, BamH1, 1.2 kb) (from Dr Gordon Peters, ICRF, London).

v-*erb*-B: pverb-B-DT (BamH1, 0.5 kb) (from D. Tannahill, Edinburgh University).

The plasmids pSPH-*ras* 2 and pMC64-10 were constructed by insertion into the vector SP64 of (respectively) the 2.9 kb SacI fragment of the human T24 Ha-*ras* gene (containing all four coding exons) or the 8.4 kb EcoRI/HindIII fragment of the human c-*myc* gene (containing all three coding exons). Oncogene RNA was generated by incubation with SP6 polymerase (Melton *et al.*, 1984) under the conditions recommended by the manufacturer (Bethesda Research Laboratories(BRL), Paisley, Scotland).

*Immunoblotting*

Monolayers of cultured cells were detached by incubation in phosphate buffered saline containing 0.02% EDTA, and lysed in 100 mM sodium chloride, 10 mM Tris pH 7.5, 0.1% SDS, 1% NP40. Portions of snap frozen tumour were treated similarly. Electrophoresis in 15% polyacrylamide gels, electrophoretic blotting on nitrocellulose, and immunostaining with Y13-259 (Furth *et al.*, 1982) were conducted as previously described (Robinson *et al.*, 1986), but using an alkaline phosphatase-streptavidin system (Blu-gene, BRL) to detect the biotinylated second antibody.

### Results

*Pathology of* 'primary' *tumours*

A summary of the tumour development at the subcutaneous injection sites is given in Table I. Animals injected in groin and back showed similar features and are considered together.

CHL cells without human oncogenes never produced visible or palpable tumours, and on autopsy 5 weeks after inoculation no abnormality was detected at the injection site. 208F cells, without human oncogenes, although reputedly non-tumorigenic, consistently yielded small, slowly-growing nodules, not exceeding 5 mm diameter even after 5 weeks. Histologically these were low grade fibrosarcomas, with few mitotic figures. There was conspicuous collagen deposition around and between the tumour cells, and mast cells of presumed host origin were plentiful within the tumours. No necrosis was observed within these tumours, but cells at the tumour edge appeared to be infiltrating between adjacent adipocytes (Figure 1A).

**Table I** Pathology of fibroblast tumours expressing human oncogenes

| *Host cell* | *Transfected* onc | *Mice injected* | *Primary tumours* | *Regressing tumours* | *Progressive tumours* | *Metastasis in lung* (*L*) | *Metastasis in node* (*N*) | *Total metastases* | *Time metastases noted* (*day after injection*) |
|---|---|---|---|---|---|---|---|---|---|
| Hamster fibroblast | nil | 4 | 0 (0%) | 0 (0%) | 0 (0%) | 0 | 0 | 0 | |
| (CHL) | T24 H-*ras* | 16 | 16 (100%) | 5 (31.3%) | 11 (68.8%) | 3 | 3 | 6 (37.5%) | L: 8, 13, 13<br>N: 18, 25, 35 |
| Rat fibroblast | nil | 7 | 0 (0%)[a] | 0 (0%) | 0 (0%) | 0 | 0 | 0 | |
| (208F) | T24 Ha-*ras* | 31 | 29 (93.5%) | 5 (16.1%) | 24 (77.4%) | 6 | 5 | 8 (25.8%) | L: 8, 11, 11} 14, 14} 14}<br>N: 11, 11} 14, 14} 14} |
| | Ha-*ras-1* | 15 | 14 (93.3%) | 2 (13.3%) | 12 (80%) | 0 | 2 | 2 (13.3%) | N: 12, 12 |
| | c-*myc* | 25 | 13 (52.0%)[b] | 0 (0%) | 13 (52%) | 1 | 1 | 2 (8.0%)[c] | L: 22<br>N: 67 |

All bracketed figures are percentages of total injected animals. [a]As indicated in the text all animals showed small nodules at the injection site, not exceeding 0.5 cm in diameter; [b]Take rate differs significantly from rat fibroblast tumours bearing c-Ha-*ras* ($P<0.01$) or T24 Ha-*ras* ($P<0.0001$; Fisher's exact test); [c]Metastasis rate differs significantly from rat fibroblast tumours bearing T24 Ha-*ras* if scored at 14 days, whether all injected animals ($P<0.01$) or only those with primary tumours ($P<0.05$) are considered.

**Figure 1** Growth and regression of rodent fibroblasts in immune suppressed mice. (a): An indolent s.c. nodule produced by injection of the 208F aneuploid rat line. Infiltration between adipocytes is seen (arrow). (b) Nascent collagen fibres at the membrane of CHL cells transfected with T24 Ha-*ras*. (c): Aggressive growth of 208F cells transfected with T24 Ha-*ras*. Muscle fibres in process of destruction by the tumour are seen (arrows). (d): Fat necrosis at site of regressing tumour, after injection of CHL cells transfected with T24 Ha-*ras*. (a, c, d × 160; H & E. b × 30,000; uranyl acetate and lead citrate).

CHL cells bearing the T24 Ha-*ras* gene yielded rapidly growing tumours in all animals, reaching diameters of over 1 cm within 2 weeks. The tumours infiltrated, and sometimes ulcerated, the overlying skin but were tethered to underlying connective tissue only rarely. Histologically these were aggressive fibrosarcomas with a high mitotic rate and little collagen between cells although nascent collagen bundles were identified on electron microscopy (Figure 1B). Zones of necrosis were conspicuous in the centre of the tumours but mast cells were not identified. Cells at the tumour periphery infiltrated around and within the striated muscle fibres of the panniculus carnosus, apparently destroying them (Figure 1C). Infiltration into adjacent fat and around epidermal appendages was also evident, but a mantle of mononuclear cells of uncertain histogenesis was often observed running circumferentially around the deep margin of the tumour. Several tumours showed extensive infiltration by neutrophil polymorphs, and regression was obvious in a proportion harvested between 13 and 35 days. Histologically, such clearly regressing tumours consisted of masses of fat necrosis with heavy neutrophil polymorph infiltration (Figure 1D). Despite this evidence of regression in some animals, the majority of tumour-bearing animals bore progressively enlarging tumours at the time of autopsy.

208F cells bearing T24 Ha-*ras* genes produced rapidly enlarging fibrosarcomas of similar morphology to the above (Figure 2A) and with a similar proportion showing regression. Similarly, 208F cells expressing the normal human Ha-*ras*-1 gene produced rapidly growing, potentially lethal tumours, although these showed less necrosis at similar size than the tumours bearing the mutated oncogenes. 208F cells bearing human c-*myc* also produced growing tumours, but in a significantly smaller proportion of animals than after injection of *ras*-bearing cells. In seven of the animals the tumours were aggressive fibrosarcomas (Figure 2B), but in the remaining six the morphology was intermediate between this and the indolent pattern of 208F cells alone. All these tumours were substantially larger than those produced by 208F cells alone, as observed at autopsy at closely similar times after inoculation. However, they grew less rapidly than the more aggressive variants, reached smaller diameters at the time of autopsy, and did not contain central necrotic areas.

**Figure 2** Aggressive fibrosarcomas at site of injection of 208F cells transfected with T24 Ha-*ras* (a) and c-*myc* (b). Numerous mitotic figures are present in both, but in (b) apoptotic bodies are also conspicuous (arrows), intimately admixed with the dividing cells. ( × 500; H & E).

### *Oncogene expression in aggressive fibrosarcomas*

Expression of c-*myc*, Ha-*ras*, and nine proto-oncogenes was assessed in examples of the aggressive fibrosarcomas, harvested within 2 weeks of inoculation (Table II and Figure 3). In tumours bearing the mutated human T24 Ha-*ras* gene, Ha-*ras* transcripts were present at levels close to those in the originally injected cells. Thus, in both CHL and 208F cells containing the T24 Ha-*ras* gene, levels of transcript were estimated at around 0.8% of total cellular mRNA, between 20- and 53-fold greater than the untransfected parent cells.

**Figure 3** Hybridisation of $^{32}P$ probes to RNA from parental fibroblasts (CHL, 208F), their derivative cell lines *in vitro* (+T24 *ras*, +*myc*) and the tumours engendered by these (+T24 *ras t*, +*myc t*). Thirteen filters were prepared identically, by spotting 5 μg total RNA from the sources shown. The filters were also spotted with 2 ng plasmid DNA: for each filter the plasmid contained the sequence to be used as hybridisation probe. After hybridisation (as in **Materials and methods**), the filters were washed twice in 2 × SSC, 0.1% SDS at 20°C and then for a further 2 h at 65°C in 0.1 × SCC, 0.1% SDS (filters 1, 2, 10, 13) or in 1 × SSC, 0.1% SDS (filters 3–9, 11, 12). The lower stringency was chosen for these latter filters to allow for the less perfect homology between the probe and target sequences. Radiographic exposure time was adjusted so that approximately equal signals were obtained from the plasmid DNA spots, with the exception of filter 12, which was exposed for the same time as filter 13. These two filters were included to confirm that all the spots were equally loaded in terms of total RNA.

**Table II** Quantitative expression of human oncogenes in fibroblasts *in vitro* and *in vivo*

| *RNA source* | | onc *RNA* | | |
|---|---|---|---|---|
| *Cell* | *Inserted* onc | *Type* | % *Total RNA* | % *mRNA* |
| CHL *in vitro* | nil | Ha-*ras* | 0.0003 | 0.013 |
| CHL *in vitro* | T24 Ha-*ras* | Ha-*ras* | 0.016 | 0.80 |
| CHL *in vivo* | T24 Ha-*ras* | Ha-*ras* | 0.015 | 0.75 |
| 208F *in vitro* | nil | Ha-*ras* | 0.0009 | 0.045 |
| 208F *in vitro* | T24 Ha-*ras* | Ha-*ras* | 0.018 | 0.90 |
| 208F *in vivo* | T24 Ha-*ras* | Ha-*ras* | 0.017 | 0.85 |
| 208F *in vitro* | nil | *myc* | 0.0006 | 0.03 |
| 208F *in vitro* | c-*myc* | *myc* | 0.011 | 0.55 |
| 208F *in vivo* | c-*myc* | *myc* | 0.009 | 0.45 |

Methods for calculating *onc* RNA as % total of mRNA, by calibration *versus* purified *onc* RNA, are given in the text.

The aggressive fibrosarcomas which derived from 208F cells bearing the human c-*myc* gene showed a similar pattern, although the steady state levels of *myc* transcript were nearer 0.5% of total mRNA, around 18-fold more than the untransfected cells. 208F cells bearing the normal human Ha-*ras*-1 gene were not studied quantitatively for oncogene mRNA, but semiquantitative dot blots (not shown) indicated that Ha-*ras* transcripts were rather more abundant than in the cells expressing the mutated gene. Further, immunoblots of extracts from these cells and the corresponding tumours showed that the expressed p21*ras* had the faster electrophoretic mobility expected of the product of the normal gene (Figure 4). The immunoblots also confirmed that the cells bearing the normal human proto-oncogene expressed much more p21*ras* than the parental fibroblasts (in which p21*ras* was barely detectable by the methods used) and to at least as great a degree as the cells expressing the T24 Ha-*ras* gene.

In contrast to these high levels of expression of the inserted human oncogenes, several other oncogenes tested (including *src*, *erb-B*, *fes*, *myb*, *int*-2 and Ki-*ras*) showed low or barely detectable levels of expression in the aggressive tumours themselves, the cells transfected with human c-*myc* and Ha-*ras* prior to animal injection, and the parental untransfected cells (Figure 3). Moderate expression of c-*sis* was observed in both parental fibroblast lines and their *ras*- and *myc*-expressing derivatives. In contrast, substantially elevated expression of *abl* and *fos* was noted in the transfected cells relative to both their parental untransfected fibroblasts, regardless of whether the transfected gene was human c-*myc* or T24 Ha-*ras*.

### *Metastases from* ras- *and* myc-*expressing tumours*

In around one third of animals in which tumours expressing T24 Ha-*ras* grew at the inoculation site, there was evidence of either blood- or lymph-borne metastasis (Table I). Blood-borne metastases were all observed within 14 days, as were the majority of the lymphatic metastases. Of the animals injected with cells expressing normal human Ha-*ras*-1, two developed metastases, both in lymph nodes, but these also were detected within 14 days of injection. Of the 13 animals in which *myc*-expressing cells produced a progressively growing 'primary' tumour, two showed metastases, one by lymphatics, the other (presumably haematogenous ) in the lung. In one of these animals the 'primary' tumour showed the aggressive type of histology. The total number of animals with metastatic tumours was small, and the lower overall yield with *myc*-expressing tumours, although suggestive, is

**Figure 4** Polyacrylamide gel electrophoresis of proteins from fibroblasts growing *in vitro* and *in vivo*. The left hand panel is a kenacid-stained gel, the right hand an immunoblot of a similarly-loaded gel, incubated with the anti p21$^{ras}$ monoclonal Y13 259, and detected with alkaline phosphatase linked to streptavidin-biotin complex. The tracks show proteins from (1) 208F cells transfected with normal human Ha-*ras*-1, growing *in vivo* as an aggressive tumour, (2) 208F cells transfected with T24 Ha-*ras*, *in vivo*, (3) CHL cells, with T24 Ha-*ras*, *in vitro*, (4) untransfected parental 208F cells, (5) as (1) but growing *in vitro*, (6) as 2, but *in vitro*. The mobility of mol. wt standards is shown on the right. Both *in vitro* and as a tumour *in vivo*, 208F with normal Ha-*ras*-1 appears to express slightly more p21$^{ras}$ than 208F with T24 Ha-*ras*.

not statistically significant. Nonetheless, the metastases in both animals with *myc*-expressing secondary tumours were detected substantially later than all the metastases from *ras*-expressing tumours by the corresponding route. If metastases detected by two weeks only are considered, the difference in incidence between T24 Ha-*ras* and c-*myc* expressing tumours is significant (Table I).

Morphologically, blood-borne metastases from the *ras*-bearing tumours had similar morphology, regardless of whether the original transfected cell was of CHL or 208F type. Such metastases were found only in the lung, and clearly emanated from small branches of the pulmonary artery, filled with tumour cells (Figure 5A). Lymphatic metastases were found in the para-iliac and para-aortic nodes in animals injected subcutaneously in the groin, and in the axillary nodes in animals injected in the back. The affected lymph nodes were usually entirely occupied by tumour cells, but in some there was evidence of partial colonisation, commencing in the subcapsular sinus (Figure 5B).

In the single example of lymphatic metastasis from *myc*-expressing 208F cells, the histology was similar to that in the *ras*-expressing tumours, but the pulmonary metastasis was unusual: a single small intra-pulmonary tumour nodule was associated with malignant pleural effusion.

**Figure 5** Spread of 208F cells expressing T24 Ha-*ras* to distant sites. (a). Metastasis in small peribronchial vessels in lung. (b). Growth in subcapsular sinus of lymph node. ( x 160; H & E).

*Cell turnover in aggressive* myc- *and Ha*-ras-*expressing tumours*

The small nodules engendered by 208F cells, and the rapidly growing tumours of 208F cells expressing human *myc* and *ras*, were studied histologically in more detail, in order to compare the effects of oncogenes on patterns of cell turnover in these tumours (Figure 6). Mitotic and apoptotic cells were counted within the clearly viable, non-necrotic regions, avoiding any areas in which infiltration by inflammatory cells or extravasation of red cells was conspicuous. In the small, indolent fibrosarcomas engendered by 208F cells alone mitotic rates were consistently low. In animals killed at 33 days single cell loss by apoptosis was also infrequent, but larger numbers of apoptotic cells were observed in one animal killed at 14 days. The oncogene-expressing tumours were studied only at 9–14 days after injection. 208F cells bearing T24 Ha-*ras* produced tumours with high mitotic rates ($\sim$10 fold more than parental 208F cells at the same time after injection) but low apoptotic rates. Tumours expressing the normal Ha-*ras*-1 gene had lower mitotic rates, but apoptosis was more frequent. In the more aggressive c-*myc* expressing tumours harvested at around the same time after injection both mitotic and apoptotic rates were high.

**Figure 6** Incidence of mitosis (●) and apoptosis (○) in the viable portions of tumours expressing normal human Ha-*ras*-1, T24 Ha-*ras*, and c-*myc*, or in the nodules engendered by the parental 208F fibroblasts. All tissues were harvested between 9 and 21 days after injection, with the exception of the 208F nodules marked ●–, ○–, which were harvested at 33 days. For each animal the mean incidence of mitosis and apoptosis in 10 high power fields was measured. There are significant differences in incidence of mitosis between Ha-*ras*-1 and both T24 Ha-*ras* and c-*myc* tumours ($P<0.001$; Student's *t* test). Significant differences in incidence of apoptosis exist between T24 Ha-*ras* and both Ha-*ras*-1 and c-*myc* tumours ($P<0.001$) and also between Ha-*ras*-1 and c-*myc* tumours ($P<0.02$).

### Discussion

Several groups have reported the ability of *ras* genes (with and without mutation) to confer metastatic activity on fibroblasts (Spandidos & Wilkie, 1984; Thorgeirsson *et al.*, 1985; Muschel *et al.*, 1985; Bradley *et al.*, 1986; Liotta, 1986) although the assays used have focussed almost exclusively on haematogenous tumour spread. Here we present evidence that high expression of the mutated T24 Ha-*ras*-1 gene confers aggressive properties, to an indistinguishable degree, on both early and late passage fibroblasts. High expression of the normal human Ha-*ras*-1 proto-oncogene is also associated with rapidly growing tumours capable of metastasis. In contrast, high expression of *myc*, in late passage fibroblasts, is associated with tumours which grow less aggressively as measured in terms of primary 'take' rate, growth rate, histology at the injection site, and the speed of development and proportion of animals affected by metastasis.

We have found no evidence that blood-borne and lymphatic metastases are differentially affected by *myc* or *ras* genes. Clearly, this study carries the usual strictures on interpretation of the metastatic process (Mareel & van Roy, 1986). Thus, although we attempted to inoculate cells into the subcutaneous tissue spaces, we cannot exclude the possibility that some cells entered damaged veins or lymphatics at the time of injection, rather than entering these in the course of tumour growth. It has frequently been argued, however, that even should such direct implantation into vessels have occurred, the development of tumours at distant sites still depends upon many of the factors essential for *bona fide* metastasis of true primary tumours (Nicolson & Poste, 1983; Liotta, 1986).

In experiments of this design it is not possible to be completely certain that expression of the inserted human oncogene is solely responsible for the malignant phenotype observed. One trivial explanation would be that the different types of growth behaviour observed are entirely coincidental features of the particular cell clones studied. This seems improbable, as high expression of T24 Ha-*ras* engenders tumours with rather similar behaviour in entirely different lines – CHL and 208F in this study, and fibroblasts of other origins elsewhere (Muschel *et al.*, 1985; Bradley *et al.*, 1986; Pozzatti *et al.*, 1986). A second possibility is that the new cell behaviour appearing after transfection is due to the viral enhancers in the transfected plasmids rather than the oncogenes themselves. The role of enhancers in carcinogenesis has been emphasised (Chichutek & Duesberg, 1986; Lang & Spandidos, 1986). Control experiments have already shown, however, that cells transfected with the enhancers but not the oncogenes underwent senescence, demonstrated no features of transformation *in vitro*, and apparently failed to generate progressive tumours (Spandidos & Wilkie, 1984). A third and less readily dismissed possibility is that the aggressive features observed are due to genetic changes related to the oncogene expression only in a permissive sense, but occuring during the process of selection for growth *in vitro* and *in vivo*. Substantial cell selection necessarily occurs between the time of transfection and animal inoculation. There is also good evidence that growth of inoculated cells *in vivo* can be associated with major changes in phenotype (Nicolson & Poste, 1983) and oncogene arrangement (Winter & Perucho, 1986). Karyotype analysis has shown that the *ras*-expressing CHL cells show several chromosomal changes as compared to the parent, untransfected cells which have a normal, diploid karyotype (Spandidos *et al.*, 1985*a*). The 208F karyotype is also hyperdiploid. However, *ras*-expressing fibroblasts have been found to metastasise without showing obvious progressive karyotypic changes *in vivo* (Muschel *et al.*, 1986), and we have confirmed here that levels of expression of both the transfected oncogenes and several proto-oncogenes remain remarkably constant during the growth of aggressive fibrosarcomas *in vivo*.

It is of interest to compare the level of expression of *myc* and *ras* genes in the transfected fibroblasts used here with that in authentic human tumours. Although absolute values of oncogene mRNA are seldom reported for the cells of human tumours, the ratio of expression in neoplastic *versus* control tissue is well documented. Thus *myc* expression may show elevation in fresh leukaemic cells of 10–50 fold over that in resting lymphocytes (Rothberg *et al.*, 1984) and of 17 fold between the most and least expressing examples of prostatic adenocarcinoma (Fleming *et al.*, 1986). Even in the course of non-neoplastic cell activation, *myc* expression may rise up to 40 fold (Kelly *et al.*, 1983). The *myc* expression in our transfected fibroblasts of around 20 fold above the parental level is thus well within this range. Increased expression of *ras*, although usually at more moderate levels in both neoplastic and normal cells (Goyette *et al.*, 1983; Slamon *et al.*, 1984; Spandidos & Agnantis, 1984) has been reported in benign colonic neoplasms as high as 30 fold more than normal epithelium (Spandidos & Kerr, 1984).

An unexpected finding in this study was the observation that c-*abl* and c-*fos* were highly expressed in fibroblasts transfected with recombinant plasmids carrying the T24 Ha-*ras* or c-*myc* genes. To our knowledge this is the first demonstration of alteration in expression of endogenous c-*oncs* after insertion of extraneous oncogenes in high expression vectors although accumulation of c-*fos* protein has been induced in 3T3 fibroblasts by microinjection of p21*ras* (Stacey *et al.*, 1987). One implication of this observation is that all the tumorigenic cell lines investigated here express oncogenes of both nuclear- and membrane-associated groups (*fos* or *myc* and *abl* or *ras*). Hence the generalisation that tumorigenicity requires co-activation of complementary oncogene groups (Land *et al.*, 1983; Ruley, 1983; Weinberg, 1985) may apply even to cells transfected with single extraneous oncogenes. We have no direct evidence on the nature of the relationship between expression of the transfected *myc* and *ras* genes and the activation of the endogenous c-*abl* and c-*fos*. Enhanced expression of receptors for $\alpha$ or $\beta$ TGF, and increased release of TGFs, have been recorded in *ras* and *myc* expressing cells however (Ozanne *et al.*, 1982; Heldin & Westermark, 1984; Anzano *et al.*, 1985; Pragnell *et al.*, 1985; Spandidos, 1985), and activation of c-*fos* is known to be dependent upon a wide spectrum of growth factors (Kruijer *et al.*, 1985; Colletta *et al.*, 1986). It appears unlikely that this activation of c-*abl* and c-*fos* could be solely responsible for the aggressive growth of the transfected lines, however, since levels of expression in the transfected cells were similar for both *ras* and *myc* bearing transfectants, despite the differences in behaviour of these cells *in vivo*.

One outstanding difference between *myc*- and *ras*-expressing tumours of otherwise similar histology was the disparity in their rates and modes of cell death. It has long been appreciated that two contrasting modes of death can be observed in tissues: necrosis, characterised by cell swelling and rupture, and apoptosis, in which single cells shrink and fragment, but retain intact membranes, and are subject to phagocytosis by their viable neighbours (Kerr *et al.*, 1972; Wyllie *et al.*, 1980). Zones of necrosis appeared in both *ras*- and *myc*-expressing fibrosarcomas as they do in most malignant tumours of man and animals. Although there is much current interest in the cause of this, and a range of explanations is offered, including lack of oxygen and nutrient (Rotin *et al.*, 1986), inadequate production of angiogenic factors (Denekamp & Hobson, 1982; Folkman, 1986), or the effect of tumour necrosis factors (Old, 1985; Klostergaard *et al.*, 1986), it is clear that in our material, necrosis of this type appeared in the more aggressive fibrosarcomas rather than the small, indolent ones, and bore no correlation with other indices of aggression. In contrast, cell loss by apoptosis within the viable portion of the tumour, which is also a feature of most growing human and animal tumours (Wyllie, 1985), was much commoner (relative to mitosis) in *myc*-expressing tumours as compared with the generally more aggressive tumours expressing T24 Ha-*ras*. The tumours expressing normal human Ha-*ras*-1 showed an intermediate pattern. It has long been suspected that the high cell loss rates observed in many growing animal and human tumours may represent an intrinsically regulated process (Kerr *et al.*, 1972; Wyllie *et al.*, 1980). We do not know what factors within these tumours may be responsible, but it is interesting to speculate that one of the results of expression of the mutated Ha-*ras* oncogene may be to override the spontaneous deletion of cells within tumour populations, and so perhaps conserve cells with progressive deviation from normality.

In conclusion, high levels of expression of *ras* and *myc* oncogenes in rodent fibroblasts are associated with a range of new cellular features including aggressive growth, characteristic patterns of cell turnover, and transcriptional activation of endogenous proto-oncogenes. Several of these features have no obvious immediate relationship with the known sites and modes of action of *ras* p21 products (Wakelam *et al.*, 1986) or the *myc* nucleoprotein (Rabbits, 1985). The dependence of these secondary features on the high *ras* and *myc* expression can best be confirmed by use of new plasmid constructs in which oncogene expression can be modulated by extracellular agents, but the data presented here are consistent with the view that biological aggressiveness may be the result of a constellation of cellular features, engendered in different proportions by the products of different oncogenes.

We are grateful to Irene Evans and David Burns and to the staff at the Western General Hospital Animal House for technical assistance. This work was supported by a grant to AHW from the Cancer Research Campaign.

**References**

ANZANO, M.A., ROBERTS, A.B., DeLARCO, J.E. & 6 others (1985). Increased secretion of type beta transforming growth factor accompanies viral transformation of cells. *Mol. Cell. Biol.*, **5**, 242.

BRADLEY, M.O., KRAYNAK, A.R., STORER, R.D. & GIBBS, J.B. (1986). Experimental metastasis in nude mice of NIH 3T3 cells containing various *ras* genes. *Proc. Natl Acad. Sci.*, **83**, 5277.

BRODEUR, G.M., SEEGER, R.C., SCHNAB, M., VARMUS, H.E. & BISHOP, J.M. (1984). Amplification of N-*myc* in untreated human neuroblastomas correlated with advanced disease stage. *Science*, **224**, 1121.

CHIRGWIN, J.M., PRZYBYLA, A.E., MACDONALD, R.J. & RUTTER, W.J. (1979). Isolation of biologically active ribonucleic acid from sources enriched in ribonuclease. *Biochemistry*, **18**, 5294.

CICHUTEK, K. & DUESBERG, P.H. (1986). Harvey *ras* genes transform without mutant codons, apparently by truncation of a 5′ exon (exon-1). *Proc. Natl Acad. Sci. USA*, **83**, 2340.

COLLARD, J.G., VAN BECK, W.P., JANSSEN, J.W. & SCHIJVEN, J.F. (1985). Transfection by human oncogenes: concomitant induction of tumorigenicity and tumour-associated membrane alterations. *Int. J. Cancer*, **35**, 207.

COLLETTA, G., CIRAFICI, A.M. & VECCHIO, G. (1986). Induction of the c-*fos* oncogene by thyrotropic hormone in rat thyroid cells in culture. *Science*, **233**, 458.

DeBERTOLI, M.E., ABOU-ISSA, H., HALEY, B.E. & CHO-CHUNG, Y.S. (1985). Amplified expression of p21*ras* protein in hormone dependent mammary carcinomas of humans and rodents. *Biochim. biophys. res. commun.*, **127**, 699.

DENEKAMP, J. & HOBSON, B. (1982). Endothelial-cell proliferation in experimental tumours. *Br. J. Cancer*, **46**, 711.

FLEMING, W.H., HAMEL, A., MacDONALD, M. & 5 others (1986). Expression of the c-*myc* proto oncogene in human prostatic carcinoma and benign prostatic hyperplasia. Cancer Res., **46**, 1535.

FOLKMAN, J. (1986). How is blood vessel growth regulated in normal and neoplastic tissue? *Cancer Res.*, **46**, 467.

FURTH, M.E., DAVIS, L.J., FLEURDELYS, B. & SCOLNICK, E.M. (1982). Monoclonal antibodies to the p21 products of the transforming gene of Harvey murine sarcoma virus and of the cellular *ras* gene family. *J. Virol.*, **43**, 1795.

GALLICK, G.E., KURZROCK, R., KLOETZER, W.S., ALPINGHAUS, R.B. & GUTTERMAN, J.U. (1985). Expression of p21*ras* in fresh primary and metastatic human colorectal tumours. *PNAS*, **82**, 1795.

GOYETTE, M., PETROPOULOS, C.J., SHANK, P.R. & FAUSTO, N. (1983). Expression of a cellular oncogene during liver regeneration. *Science*, **219**, 510.

HANNA, N. & SCHNEIDER, M. (1983). Enhancement of tumor metastasis and suppression of NK cell activity by B-estradiol treatment. *J. Immunol.*, **130**, 974.

HAY, J.H., MORTEN, J.E.N., CLARKE, B. & SWINTON, J. (1985). The suitability of immunosuppressed mice, kept in a standard animal unit, as recipients of human tumour xenografts. *Lab. Animals*, **19**, 119.

HELDIN, C.H. & WESTERMARK, B. (1984). Growth factors: Mechanism of action and relation to oncogenes. *Cell*, **37**, 9.

JOHNSON, P.W., BAUBOCK, C. & RODER, J.C. (1985). Transfection of a rat cell lines with the v-Ki-*ras* oncogene is associated with enhanced susceptibility to natural killer cell lysis. *J. exp. med.*, **162**, 1732.

KASID, A., LIPPMAN, M.E., PAPAGEORGE, A.G., LOWY, D.R. & GELLMAN, E.P. (1985). Transfection of v-*ras* DNA into MCF-7 human breast cancer cells bypasses dependence on estrogen for tumorigenicity. *Science*, **228**, 725.

KELLY, K., COCHRAN, B.H., STILES, C.D. & LEDER, P. (1983). Cell specific regulation of the c-*myc* gene by lymphocyte mitogens and platelet-derived growth factor. *Cell*, **35**, 603.

KERR, J.F.R., WYLLIE, A.H. & CURRIE, A.R. (1972). Apoptosis: A basic biological phenomenon with wide-ranging implications in tissue kinetics. *Br. J. Cancer*, **26**, 239.

KLEIN, G. & KLEIN, E. (1986). Conditioned tumorigenicity of activated oncogenes. *Cancer Res.*, **46**, 3211.

KLOSTERGAARD, J., FOSTER, W.A., HAMILTON, D.A., TURPIN, J. & LOPEZ-BERESTEIN, G. (1986). Effector mechanisms of human monocyte-mediated tumor cytotoxicity *in vitro*: Biochemical, functional and serological characterization of cytotoxins produced by peripheral blood monocytes isolated by counterflow elutration. *Cancer Res.*, **46**, 2871.

KRIS, R.M., AVIVI, A., BAR-ELI, M., ALON, Y., CARMI, P., SCHLESSINGER, J. & RAZ, A. (1985). Expression of Ki-*ras* oncogene in tumor cell variants exhibiting differing metastatic capabilities. *Int. J. Cancer*, **35**, 227.

KRUIJER, W., SCHUBERT, D. & VERMA, I.M. (1985). Induction of the proto-oncogene *fos* by nerve growth factor. *Proc. Natl Acad. Sci. USA*, **82**, 7330.

KURZROCK, R., GALLICK, G.E. & GUTTERMAN, J.U. (1986). Differential expression of p21*ras* gene products among histological subtypes of fresh primary lung tumours. *Cancer Res.*, **46**, 1530.

LAND, H., PARADA, L.F. & WEINBERG, R.A. (1983). Tumorigenic conversion of primary embryo fibroblasts requires at least two cooperating oncogenes. *Nature*, **304**, 596.

LANG, J.C. & SPANDIDOS, D.A. (1986). The structure and function of eukaryotic enhancer elements and their role in oncogenesis. *Anticancer Res.*, **6**, 437.

LIOTTA, L.A. (1986). Tumor invasion and metastasis – role of extracellular matrix. *Cancer Res.*, **46**, 1.

MANIATIS, T., FRITSCH, E.F. & SAMBRROK, J. (1982). *Molecular cloning, a laboratory manual.* Cold Spring Harbor Laboratory, p. 202.

MAREEL, M.M. & VAN ROY, F.M. (1986). Are oncogenes involved in invasion and metastasis? *Anticancer Res.*, **6**, 419.

MARSHALL, C.J., VOUSDEN, K. & OZANNE, B. (1985). The involvement of activated *ras* genes in determining the transformed phenotype. *Proc. Roy. Soc.* (*Lond*)., **B 226**, 99.

MELTON, D.A., KRIEG, P.A., REBAGLIATI, M.R., MANIATIS, T., ZINN, K. & GREEN, M.R. (1984). Efficient *in vitro* synthesis of biologically active RNA and RNA hybridisation probes from plasmids containing a bacteriophage SP6 promoter. *Nucleic Acids Res.*, **12**, 7035.

MUSCHEL, R.J., NAKAHARA, K., CHU, E., POZZATTI, R. & LIOTTA, L.A. (1986). Karyotypic analysis of diploid or near-diploid metastatic Harvey *ras* transformed rat embryo fibroblasts. *Cancer Res.*, **46**, 4104.

MUSCHEL, R.J., WILLIAMS, J.E., LOWY, D.R. & LIOTTA, L.A., (1985). Harvey *Ras* induction of metastatic potential depends upon oncogene activation and the type of recipient cell. *Am. J. Path.*, **121**, 1.

NICOLSON, G. & POSTE, G. (1983). Tumor implantation and invasion at metastatic sites. *Int. Rev. Exp. Path.*, **25**, 78.

OLD, L.J. (1985). Tumor necrosis factor. *Science*, **230**, 630.

OZANNE, B., WHEELER, T. & KAPLAN, P.L. (1982). Cells transformed by RNA and DNA tumor viruses produce transforming factors. *Fed. Proc.*, **41**, 3004.

POZATTI, R., MUSCHEL, R., WILLIAMS, J. & 4 others (1986). Primary rat embryo cells transformed by one or two oncogenes show different metastatic potentials. *Science*, **232**, 223.

PRAGNELL, I.B., SPANDIDOS, D.A. & WILKIE, N.M. (1985). Consequences of altered oncogene expression in rodent cells. *Proc. Roy. Soc.* (*Lond*), **B 226**, 107.

QUADE, K. (1979). Transformation of mammalian cells by avian myelocytomatosis virus and avian erythroblastosis virus. *Virology*, **98**, 461.

RACKER, E., RESNICK, R.J. & FELDMAN, R. (1985). Glycolysis and methylaminosiobutyrate uptake in Rat-1 cells transfected with *ras* or *myc* oncogenes. *Proc. Natl Acad. Sci. USA*, **82**, 3535.

RABBITS, T.H. (1985). The c-*myc* proto-oncogene: Involvement in chromosomal abnormalities. *Trends in Genetics*, **1**, 327.

ROBINSON, A., WILLIAMS, A.R.W., PIRIS, J., SPANDIDOS, D.A. & WYLLIE, A.H. (1986). Evaluation of a monoclonal antibody to *ras* peptide, RAP-5, claimed to bind preferentially to cells of infiltrating carcinomas. *Br. J. Cancer*, **54**, 877.

ROSEN, N., REYNOLDS, C.P., THIELE, C.J., BIEDLER, J.L. & ISRAEL, M.A. (1986). Increased N-*myc* expression following progressive growth of human neuroblastoma. *Cancer Res.*, **46**, 4139.

ROTHBERG, P.G., ERISMAN, M.D., DIEHL, R.E., ROVIGATTI, U.G. & ASTRIN, S.M. (1984). Structure and expression of the oncogene c-*myc* in fresh tumour material from patients with hematopoietic malignancies. *Mol. Cell. Biol.*, **4**, 1096.

ROTIN, D., ROBINSON, B. & TANNOCK, I.F. (1986). Influence of hypoxia and an acid environment on the metabolism and viability of cultured cells: Potential implications for cell death in tumors. *Cancer Res.*, **46**, 2821.

RULEY, H. (1983). Adenovirus early region 1A enables viral and cellular transforming genes to transform primary cells in culture. *Nature*, **304**, 602.

SLAMON, D.J., DEKERNION, J.B., VERMA, I.M. & CLINE, M.J. (1984). Expression of cellular oncogenes in human malignancies. *Science*, **224**, 227.

SPANDIDOS, D.A. (1985). Mechanisms of carcinogenesis: The role of oncogenes, transcriptional enhancers and growth factors. *Anticancer Res.*, **5**, 485.

SPANDIDOS, D.A. & AGNANTIS, N.J. (1984). Human malignant tumours of the breast as compared to their respective normal tissue have elevated expression of the Harvey *ras* oncogene. *Anticancer Res.*, **4**, 269.

SPANDIDOS, D.A., FRESHNEY, M. & WILKIE, N.M. (1985*a*). Heterogeneity of cell lines derived after transformation of early passage rodent cells by the Ha-*ras* 1 human oncogene. *Anticancer Res.*, **5**, 387.

SPANDIDOS, D.A., HARRISON, P.R. & PAUL, J. (1981). Transfer and expression of herpes simplex virus thymidine kinase and human globin genes in mammalian cells studied by spot hybridisation assays. *Bioscience Rep.*, **1**, 911.

SPANDIDOS, D.A. & KERR, I.B. (1984). Elevated expression of the human *ras* oncogene family in premalignant and malignant tumours of the colorectum. *Br. J. Cancer*, **49**, 681.

SPANDIDOS, D.A., LAMONTHE, A. & FIELD, J.K. (1985*b*). Multiple transcriptional activation of cellular oncogenes in human head and neck solid tumours. *Anticancer Res.*, **5**, 221.

SPANDIDOS, D.A. & WILKIE, N.M. (1984). Malignant transformation of early passage rodent cells by a single mutated human oncogene. *Nature*, **310**, 469.

STACEY, D.W., WATSON, T., KUNG, H.-F. & CURRAN, T. (1987). Microinjection of transforming *ras* protein induces c-*fos* expression. *Mol. Cell. Biol.*, **7**, 523.

STEEL, G.G., COURTENAY, V.D. & ROYSTON, A.Y. (1978). Improved immune suppression techniques for the xenografting of human tumours. *Br. J. Cancer*, **37**, 224.

THORGEIRSSON, U.P., TURPEENNIEMI-HUJANEN, T., WILLIAMS, J.E. & 4 others (1985). NIH/3T3 cells transfected with human tumor DNA containing activated *ras* oncogenes express the metastatic phenotype in nude mice. *Mol. Cell. Biol.*, **5**, 259.

TRIMBLE, W.S., JOHNSON, P.W., HOZUMIC, N. & RODER, C. (1986). Inducible cellular transformation by a metallothionein-*ras* hybrid oncogene leads to natural killer cell susceptibility. *Nature*, **321**, 782.

WAKELAM, M.J.O., DAVIES, S.A., HOUSLAY, M.D., McKAY, I., MARSHALL, C.J. & HALL, A. (1986). Normal $p21^{N\text{-}ras}$ couples bombesin and other growth factor receptors to inositol phosphate production. *Nature*, **323**, 173.

WEINBERG, R.A. (1985). The action of oncogenes in the cytoplasm and nucleus. *Science*, **230**, 770.

WILLIAMS, A.R.W., PIRIS, J., SPANDIDOS, D.A. & WYLLIE A.H. (1985). Immunohistochemical detection of the *ras* oncogene p21 product in an experimental tumour and in human colorectal neoplasms. *Br. J. Cancer*, **52**, 687.

WINTER, E. & PERUCHO, N. (1986). Oncogene amplification during tumorigenesis of established rat fibroblasts reversibly transformed by activated human *ras* oncogenes. *Mol. Cell. Biol.*, **6**, 2562.

WYLLIE, A.H. (1985). The biology of cell death in tumours. *Anticancer Res.*, **5**, 131.

WYLLIE, A.H., KERR, J.F.R. & CURRIE, A.R. (1980). Cell death: The significance of apoptosis. *Int. Rev. Cytol.*, **68**, 251.

ZAHN, X. & GOLDFARB, M. (1986). Growth factor requirements of oncogene-transformed NIH 3T3 and Balb/c 3T3 cells cultured in defined media. *Mol. Cell. Biol.*, **6**, 3541.